# Exploring the Efficacy of the Effortful Swallow Maneuver for Improving Swallowing in People With Parkinson Disease—A Pilot Study

**DOI:** 10.1016/j.arrct.2023.100276

**Published:** 2023-06-30

**Authors:** Pooja Gandhi, Melanie Peladeau-Pigeon, Michelle Simmons, Catriona M. Steele

**Affiliations:** a Swallowing Rehabilitation Research Laboratory, KITE Research Institute—University Health Network, Toronto, Canada; b Rehabilitation Sciences Institute, University of Toronto, Toronto, Canada

**Keywords:** Deglutition, Deglutition disorders, Dysphagia, Effortful swallow, Parkinson disease, Rehabilitation, Treatment outcomes, Videofluoroscopy

## Abstract

**Objectives:**

To determine the immediate (compensatory) and longer term (rehabilitative) effect of the effortful swallow (ES) maneuver on physiological swallowing parameters in Parkinson disease.

**Design:**

Virtual intervention protocol via Microsoft Teams with pre- and post-videofluoroscopic swallowing studies.

**Setting:**

Outpatient hospital setting, with intervention performed virtually.

**Participants:**

Eight participants (median age 74 years [63-82])with Parkinson disease (years post onset 3-20) with a Hoehn and Yahr scale score between 2 and 4 (N=8).

**Interventions:**

ES maneuver, initiated using a maximum effort isometric tongue-to-palate press, with biofeedback provided using the Iowa Oral Performance Instrument. The protocol included 30 minute sessions twice daily, 5 days/week for 4 weeks.

**Main Outcome Measures:**

Penetration-Aspiration Scale scores, time-to-laryngeal-vestibule-closure, total pharyngeal residue, and pharyngeal area at maximum constriction as seen on lateral view videofluoroscopy.

**Results:**

No consistent, systematic trends were identified in the direction of improvement or deterioration across Penetration-Aspiration Scale scores, time-to-laryngeal-vestibule-closure, pharyngeal area at maximum constriction, or total pharyngeal residue.

**Conclusions:**

Heterogeneous response to the ES as both a compensatory and rehabilitative technique. Positive response on the compensatory probe was predictive of positive response after rehabilitation.

Parkinson disease (PD) is among the most common neurodegenerative disorders, affecting 1%-2% of individuals above age 65, and the fastest-growing neurologic disease in terms of prevalence, related disability, and mortality.^1^, ^2^, ^3^, ^4^, ^5^ Currently, there are no neuroprotective therapies that prevent or delay PD progression.^6^ The loss of dopaminergic neurons in the substantia nigra and the reduction of dopamine concentration in the striatum^7^ leads to a wide range of clinical symptoms,^8^ including motor symptoms of tremor, bradykinesia, and rigidity.^9^ Clinical manifestations of PD also feature non-motor symptoms,^9^^,^^10^ including dysphagia (swallowing impairment), which has an estimated prevalence of ≥40%.^11^ Dysphagia is largely unresponsive to dopaminergic therapy and contributes to risk for aspiration pneumonia, malnutrition, and dehydration, even in the early stages of PD, making timely and efficient management crucial.^12^, ^13^, ^14^, ^15^, ^16^

Dysphagia management commonly includes compensatory measures (eg, postural adjustments, airway closure maneuvers, diet texture modifications),^17^^,^^18^ but these have poor patient acceptability and adherence. A recent systematic found no optimal interventions for dysphagia in PD, however, exercise-based interventions emphasizing effort and targeting improved swallowing efficiency showed promise, and visual biofeedback was beneficial.^18^ Furthermore, a growing number of studies recommend targeting the physiological mechanisms underlying swallowing impairment in exercise-based approaches to dysphagia therapy.^19^, ^20^, ^21^, ^22^ To this end, our group recently conducted a prospective study comparing swallowing safety, efficiency, timing, and kinematics in individuals with mild PD to healthy age- and sex-matched controls.^19^ We identified 2 key mechanisms of swallowing impairment in PD: (1) prolonged time-to-laryngeal-vestibule-closure (“LVC”, ie, airway protection), which is a risk for penetration-aspiration of food and liquid into the airway; and (2) reduced pharyngeal constriction, which is associated with pharyngeal residue after the swallow. Based on these findings, we undertook to evaluate a course of dysphagia intervention using the effortful swallow (ES) maneuver, combining elements of (1) exercise with effort; (2) experience dependent plasticity (ie, effect of the environment on the biological organization of the brain); (3) mechanistically targeted treatment; and (4) external biofeedback. Specifically, the ES was selected as it is understood to result in greater bolus driving forces and faster bolus transit secondary to increased amplitudes of oral and pharyngeal muscle contraction.^23^^,^^24^ Felix et al previously explored the effect of the ES on impairments in swallowing efficiency and safety in people with PD.^25^ While they reported decreased overall residue post intervention, it is important to note that the authors used clinical judgment to determine the presence/absence of post swallow residue, rather objective, instrumental methods of measurement. More recently, a systematic review by Bahia and Lowell concluded that that the ES leads to increased pressures in the oral, pharyngeal, and esophageal regions, but the functional effect of the ES in terms of swallowing safety and efficiency has not been adequately studied.^26^ They also emphasized the need for standardization of the ES instructions.

With this in mind, in this manuscript, we report preliminary data regarding the immediate (compensatory) and long-term (rehabilitative) effects of the ES in a case series of individuals with PD. We hypothesized that use and repeated practice of the ES would lead to shorter time-to-LVC and better pharyngeal constriction, with corresponding functional outcomes of reduced penetration-aspiration and reduced pharyngeal residue.

## Methods

This study received human subjects approval (CAPCR ID 21-5814). We adhered to the Strengthening the Reporting of Observational Studies in Epidemiology (STROBE) guidelines^27^ for reporting.

Participants were recruited from an outpatient clinic based on the criteria in table 1. Written consent was obtained. Prior to data collection, participants were taught the ES as a tongue-pressure emphasis technique, with the instruction to push the tongue hard against the roof of the mouth and swallow. During this visit, participants were also taught how to use the Iowa Oral Performance Instrument (IOPI) and how to thicken thin liquids as per the protocol. Data collection subsequently began with a baseline videofluoroscopic swallowing studies (VFSS) to confirm eligibility and probe the compensatory effects of the ES. Participants who displayed atypical values of prolonged time-to-LVC and/or poor pharyngeal constriction on regular effort swallows at baseline continued into a 4-week intervention with two 30-minute sessions of ES practice daily, 5 days/week. The amplitudes of pressures generated when performing the ES were registered on the IOPI and tracked on a recording sheet by the participant. A post-treatment VFSS measured rehabilitative outcomes on regular effort swallows. VFSS ratings were performed according the Analysis of Swallowing Physiology: Events, Kinematics and Timing for Use in Clinical Practice Method (https://steeleswallowinglab.ca/srrl/).

**Table 1:** Inclusion and exclusion criteria used to perform eligibility screening for participants

Inclusion Criteria	Exclusion Criteria
• At least 18 years old, • English-speaking, • Able to follow study instructions, • Neurologist confirmed diagnosis of PD, • Hoehn and Yahr scale score of 2 or 3, • Self-report of 1 or more swallowing or related symptoms: a) Difficulty with secretion management, (b) Coughing at the meal time, (c) Choking on food, (d) Respiratory infection in the past 6 months (other than COVID).	• History of head and neck cancer • Radical neck dissection (eg, anterior cervical spine surgery) or neck/oropharyngeal surgery (not excluded—tonsillectomy, adenoidectomy) • Past medical history of any neurologic disease other than PD (eg, multiple sclerosis, amyotrophic lateral sclerosis, traumatic brain injury, stroke) • Cognitive or receptive communication difficulties that precluded the participant's ability to follow study instructions provided in English. This was determined by the participant's physician prior to referring them to the study.

### Statistics

We adopted a descriptive approach to analyzing the data, by plotting the worst value for each participant per parameter per consistency on graphs comparing conditions (ie, baseline regular effort vs baseline effortful; and baseline regular effort swallows vs post-treatment regular effort swallows). Error bars were used to illustrate parameter score ranges and an estimated effect size for each comparison was calculated, by dividing the individual change in scores by the pooled group standard deviation of worst-scores across the conditions of interest. These effect size estimates were interpreted according to the guidance for interpreting Cohen's d, where d=0.2 is considered a “small” effect size, 0.5 represents a “medium” effect size, and 0.8 a “large” effect size.^28^ Finally, in addition to the direction of change, the magnitude of change was further classified based on whether scores moved from the atypical to the typical range, based on healthy reference values (https://steeleswallowinglab.ca/srrl/).^29^ Additional details regarding the study methods are available in appendix 1.

## Results

### Participants

Demographics are presented in table 2. All participants had a neurologist confirmed diagnosis of PD, with time since diagnosis ranging from 2 to 22 years, and Hoehn and Yahr Scale scores ranging from 2 to 4. All participants had self-reported swallowing concerns but none had received any prior swallowing intervention. Three participants did not qualify for the 4-week intervention trial: 2 did not show the physiological impairments of interest on the baseline VFSS, and 1 presented with cognitive impairment limiting her ability to participate in virtual treatment.

**Table 2:** Participant demographics

Participant	Age (y)	Sex	Year of PD Diagnosis	H&Y Scale Score	UPDRS Score 2	UPDRS Score 3	Medication State	Type of Diet/Level of Oral Intake at Baseline	Munich Dysphagia Test—PD Score	Status in Study
P1	74	F	2014	2	16	10	ON	Regular solids and thin liquids	Dysphagia with risk of aspiration Score: 8.73	Baseline and Post-treatment VFSS
P2	82	F	2019	2	Not available	Not available	ON	Regular solids and thin liquids	Not completed	Baseline VFSS only; did not qualify for intervention
P3	74	M	2014	3	Not available	38	ON	Regular solids and thin liquids	Dysphagia with risk of aspiration Score: 13.47	Baseline and post-treatment VFSS
P4	74	F	2011	4	Not available	54	ON	Regular solids and thin liquids	No noticeable dysphagia Score: 2.51	Baseline VFSS only; did not qualify for intervention
P5	74	M	2013	2	Not available	35/117*	ON	Regular solids and thin liquids	Dysphagia with risk of aspiration Score: 9.01	Baseline and post-treatment VFSS
P6	63	M	2016	2	18	30	ON	Regular solids and thin liquids	Dysphagia with risk of aspiration Score: 6.32	Baseline and post-treatment VFSS
P7	65	F	2000	4	Not available	61	ON	Pureed solids and thin liquids	Dysphagia with risk of aspiration Score: 9.01	Baseline and post-treatment VFSS
P8	68	M	2015	2	Not available	23	ON	Regular solids and thin liquids	Not completed	Baseline VFSS only; did not qualify for intervention

⁎ Denominator lower because rigidity was not assessed because of online assessment limitations by MD.

### Effortful swallow as a compensatory technique

**Fig 1:**
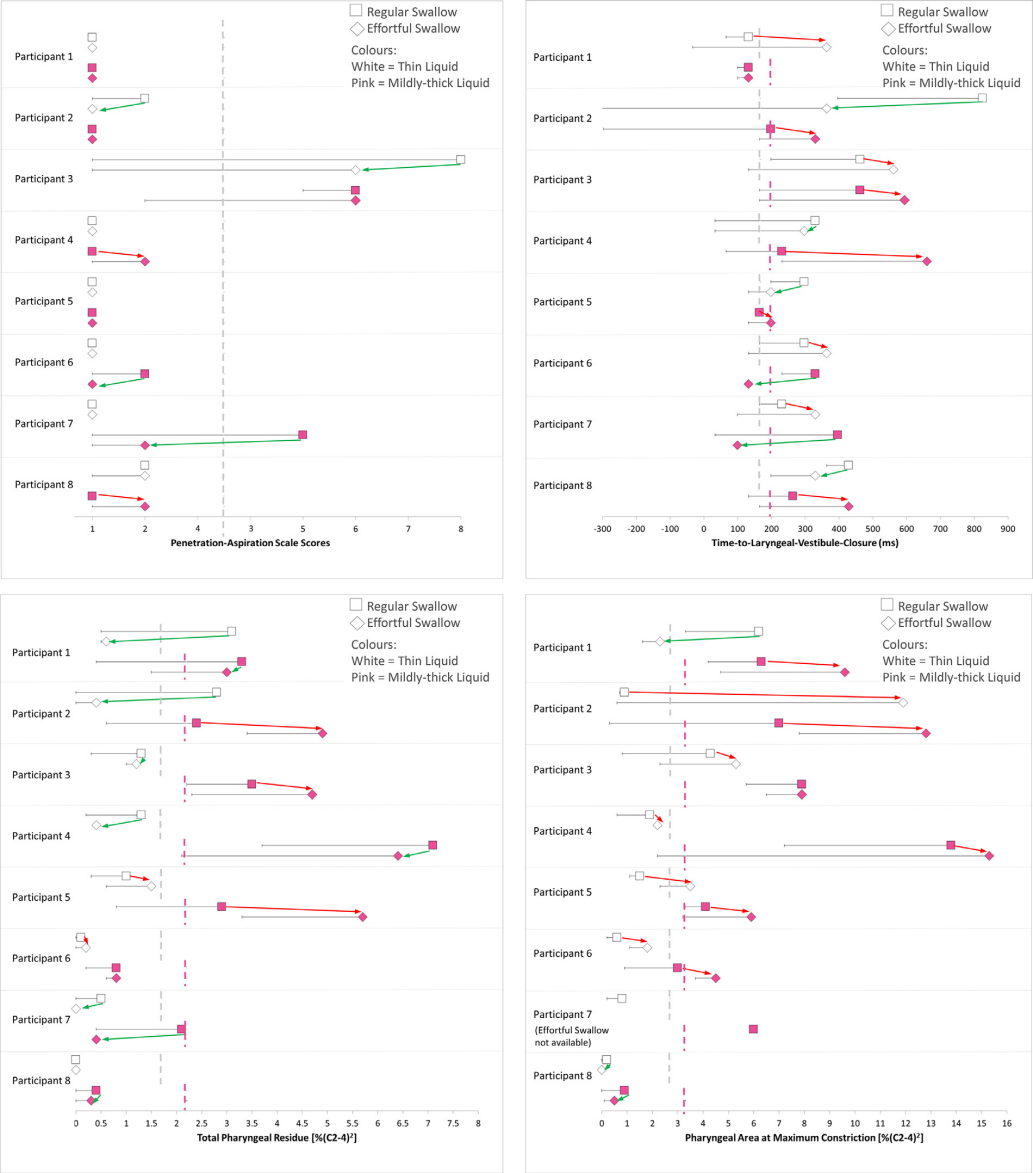
(a)–(d) Forest plots showing individual participant differences between regular swallows (squares) and effortful swallows (diamonds) at baseline. Thin liquid data are shown in white and mildly-thick liquid data in pink. The data points represent worst scores per parameter for each condition, with the error bars showing the range of scores seen across 3 task repetitions per condition. Dashed vertical lines represent the boundary between typical and atypical scores, based on healthy reference data. Green arrows indicate improvement; red arrows indicate worsening. Panel a=Penetration-Aspiration Scale scores; panel b=Time-to-Laryngeal-Vestibule-Closure; panel c=Total Pharyngeal Residue; and panel d=Pharyngeal Area at Maximum Constriction.

Figure 1 (panels a–d) provides a graphic overview of the effects of the ES maneuver when performed as a compensatory technique at the baseline VFSS. The panels are organized to enable the visualization of functional outcomes of safety and efficiency on the left side of the figure and the corresponding mechanistic parameters on the right side of the figure.

Tables 3 and 4 show the details of change during the baseline VFSS probe of the ES as a compensatory technique. The relation between changes in Penetration-Aspiration Scale (PAS)^30^ scores and time-to-LVC can be appreciated in figure 1 panels a and b. In panel 1a, participants 2 and 3 showed lower PAS scores on thin liquids in the ES condition, while participants 6 and 7 showed lower PAS scores on ES of mildly thick liquids. Of these 4 participants who demonstrated functional improvement in swallowing safety, 3 showed corresponding improvement in time-to-LVC on the respective liquid consistencies (fig 1b). Figures 1c–d capture the compensatory outcomes for swallowing efficiency, with functional (ie, total pharyngeal residue) and mechanistic (ie, PhAMPC) parameters shown side by side for each participant. In figure 1c, we see that all participants except participants 5 and 6 showed improvement in total pharyngeal residue on at least 1 consistency. Interestingly, of the 6 participants who showed improvement, only 2 showed corresponding improvement in PhAMPC (fig 1 d).

**Table 3:** Comparison of Penetration-Aspiration Scale scores between regular effort and effortful swallows at baseline (thin liquids and mildly thick liquids)

Parameter	Consistency	Participant	Regular Effort	Effortful	Direction of Change*	Magnitude of Change
Penetration-Aspiration Scale scores	Thin IDDSI level 0	P1	1	1	Unchanged	Typical –> Typical
		P2	2	1	Lower	Atypical –> Typical
		P3	8	6	Lower	Atypical –> Atypical
		P4	1	1	Unchanged	Typical –> Typical
		P5	1	1	Unchanged	Typical –> Typical
		P6	1	1	Unchanged	Typical –> Typical
		P7	1	1	Unchanged	Typical –> Typical
		P8	2	2	Unchanged	Atypical –> Atypical
	Mildly-thick IDDSI level 2	P1	1	1	Unchanged	Typical –> Typical
		P2	1	1	Unchanged	Typical –> Typical
		P3	6	6	Unchanged	Atypical –> Atypical
		P4	1	2	Higher	Typical –> Atypical
		P5	1	1	Unchanged	Typical –> Typical
		P6	2	1	Lower	Atypical –> Typical
		P7	5	2	Lower	Atypical –> Atypical
		P8	1	2	Higher	Typical –> Atypical

Abbreviation: IDDSI, International Dysphagia Diet Standardization Initiative framework.

⁎ Lower PAS scores signify change in the direction of improvement; Higher PAS scores signify change in the direction of deterioration.

**Table 4:** Comparison of continuous videofluoroscopy measures between regular effort and effortful swallows at baseline (thin liquids and mildly thick liquids)

Parameter	Consistency	Participant	Regular Effort	Effortful	Cohen's d*
Time-to-laryngeal-vestibule closure (milliseconds)	Thin IDDSI level 0	P1	132	363	−1.5
		P2	825	363	3.0
		P3	462	561	−0.6
		P4	330	297	0.2
		P5	297	198	0.6
		P6	297	363	−0.4
		P7	231	330	−0.6
		P8	429	330	0.6
	Mildly-thick IDDSI level 2	P1	132	132	0.0
		P2	198	330	−0.8
		P3	462	594	−0.8
		P4	231	660	−2.6
		P5	165	198	−0.2
		P6	330	132	1.2
		P7	396	99	1.8
		P8	264	429	−1.0
Total pharyngeal residue %(C2-C4)^2^	Thin IDDSI level 0	P1	3.1	0.6	3.1
		P2	2.8	0.4	2.9
		P3	1.3	1.2	0.1
		P4	1.3	0.4	1.1
		P5	1	1.5	−0.6
		P6	0.1	0.2	−0.1
		P7	0.5	Unable to measure	N/A
		P8	0	0	0.0
	Mildly-thick IDDSI level 2	P1	3.3	3	0.1
		P2	2.4	4.9	−1.1
		P3	3.5	4.7	−0.5
		P4	7.1	6.4	0.3
		P5	2.9	5.7	−1.2
		P6	0.8	0.8	0.0
		P7	2.1	0.4	0.7
		P8	0.4	0.3	0.0
Pharyngeal area at maximum constriction %(C2-C4)^2^	Thin IDDSI level 0	P1	6.2	2.3	1.3
		P2	0.9	11.9	−3.7
		P3	4.3	5.3	−0.3
		P4	1.9	2.2	−0.1
		P5	1.5	3.5	−0.7
		P6	0.6	1.8	−0.4
		P7	0.8	Unable to measure	N/A
		P8	0.2	0	0.1
	Mildly-thick IDDSI level 2	P1	6.3	9.6	−0.7
		P2	7	12.8	−1.3
		P3	7.9	7.9	0.0
		P4	13.8	15.3	−0.3
		P5	4.1	5.9	−0.4
		P6	3	4.5	−0.3
		P7	6	Unable to measure	N/A
		P8	0.9	0.5	0.1

Abbreviation: IDDSI, International Dysphagia Diet Standardization Initiative framework.

⁎ Positive Cohen's d score signifies change in the direction of improvement and negative Cohen's d score signifies change in the direction of deterioration.

### Effortful swallow as a rehabilitative technique

**Fig 2:**
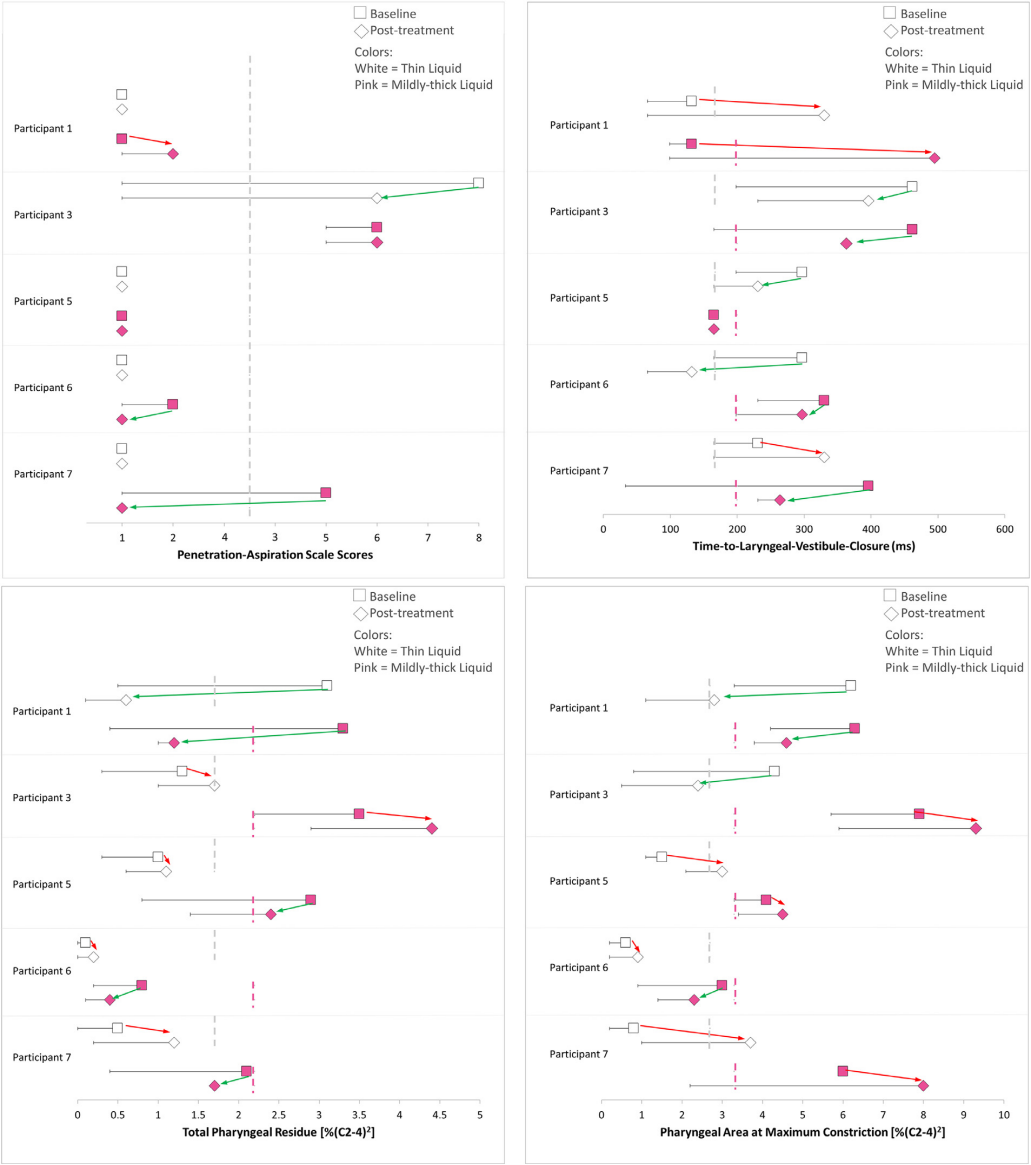
(a)–(d) Forest plots showing individual participant differences between regular swallows (squares) and effortful swallows (diamonds) after rehabilitation. Thin liquid data are shown in white and mildly-thick liquid data in pink. The data points represent worst scores per parameter for each condition, with the error bars showing the range of scores seen across 3 task repetitions per condition. Dashed vertical lines represent the boundary between typical and atypical scores, based on healthy reference data. Green arrows indicate improvement; red arrows indicate worsening. Panel a=Penetration-Aspiration Scale scores; panel b=Time-to-Laryngeal-Vestibule-Closure; panel c=Total Pharyngeal Residue; and panel d=Pharyngeal Area at Maximum Constriction.

Figure 2 (panels a–d) provides a graphic overview of the effects of 4 weeks of practicing the ES as a rehabilitative exercise, comparing baseline and post-treatment VFSS. The panels are organized to show the functional outcomes of safety and efficiency on the left and the corresponding mechanistic parameters on the right side of the figure. Tables 5 and 6 show the details of change on regular effort swallows after 4 weeks of practicing the ES as a rehabilitative techniques.

**Table 5:** Pre-post comparison of Penetration-Aspiration Scale scores after 4 weeks of practicing the Effortful Swallow (thin liquids and mildly thick liquids)

Parameter	Consistency	Participant	Baseline	Post-treatment	Change of Direction*	Magnitude of Change
Penetration-Aspiration Scale Scores	Thin IDDSI level 0	P1	1	1	Unchanged	Typical –> Typical
		P3	8	6	Lower	Atypical –> Atypical
		P5	1	1	Unchanged	Typical –> Typical
		P6	1	1	Unchanged	Typical –> Typical
		P7	1	1	Unchanged	Typical –> Typical
	Mildly-thick IDDSI level 2	P1	1	2	Higher	Typical –> Atypical
		P3	6	6	Unchanged	Atypical –> Atypical
		P5	1	1	Unchanged	Typical –> Typical
		P6	2	1	Lower	Atypical –> Typical
		P7	5	1	Lower	Atypical –> Typical

Abbreviation: IDDSI, International Dysphagia Diet Standardization Initiative framework.

⁎ Lower PAS scores signify change in the direction of improvement; higher PAS scores signify change in the direction of deterioration.

**Table 6:** Pre-post comparison of continuous videofluoroscopy measures after 4 weeks of practicing the Effortful Swallow (thin liquids and mildly thick liquids)

Parameter	Consistency	Participant	Baseline	Post-treatment	Cohen's d*
Time-to-laryngeal-vestibule closure (milliseconds)	Thin IDDSI level 0	P1	132	330	−1.8
		P3	462	396	0.6
		P5	297	231	0.6
		P6	297	132	1.5
		P7	231	330	−0.9
	Mildly-thick IDDSI level 2	P1	132	495	−2.7
		P3	462	363	0.7
		P5	165	165	0.0
		P6	330	297	0.2
		P7	396	264	1.0
Total Pharyngeal residue %(C2-C4)^2^	Thin IDDSI level 0	P1	3.1	0.6	2.9
		P3	1.3	1.7	−0.5
		P5	1	1.1	−0.1
		P6	0.1	0.2	−0.1
		P7	0.5	1.2	−0.8
	Mildly-thick IDDSI level 2	P1	3.3	1.2	1.6
		P3	3.5	4.4	−0.7
		P5	2.9	2.4	0.4
		P6	0.8	0.4	0.3
		P7	2.1	1.7	0.3
Pharyngeal area at maximum constriction %(C2-C4)^2^	Thin IDDSI level 0	P1	6.2	2.8	1.9
		P3	4.3	2.4	1.1
		P5	1.5	3	−0.9
		P6	0.6	0.9	−0.2
		P7	0.8	3.7	−1.7
	Mildly-thick IDDSI level 2	P1	6.3	4.6	0.7
		P3	7.9	9.3	−0.6
		P5	4.1	4.5	−0.2
		P6	3	2.3	0.3
		P7	6	8	−0.8

Abbreviation: IDDSI, International Dysphagia Diet Standardization Initiative framework.

⁎ Positive Cohen's d score signifies change in the direction of improvement and negative Cohen's d score signifies change in the direction of deterioration.

The relation between changes in Penetration-Aspiration Scale scores and time-to-LVC can be appreciated in figure 2, panels a and b. In panel 2a, participant 3 showed improved PAS scores on thin liquids, while participants 6 and 7 showed improved PAS scores on mildly thick liquids. All 3 of these participants showed corresponding improvement in time-to-laryngeal vestibule closure on the respective liquid consistencies.

The relation between changes in total pharyngeal residue and PhAMPC can be appreciated in figure 2, panels c-d. As shown in panel c, participants 1, 5, 6, and 7 showed lower total pharyngeal residue scores post-treatment. Of these participants, 2 showed corresponding improvement in PhAMPC on the respective liquid consistencies (fig 2d).

## Discussion

The purpose of this study was to explore the preliminary efficacy of a targeted, effort-based swallowing intervention, both as a short-term compensatory technique, and after 4 weeks of intensive practice as a rehabilitation technique in people with PD. Our findings show variability in the direction of change (improvement and deterioration) for specific parameters across participants and bolus consistencies. No systematic trends were observed when comparing the effect of the ES on thin liquid vs mildly thick liquid trials; patients with earlier vs later onset of PD; mild vs severe PD severity; or related to the subjective burden of dysphagia as reported on the Munich Dysphagia Test.^31^ While changes in either direction did not appear to be predictable, individual patients did show improvement to varying degrees on particular parameters with the ES maneuver. All participants who showed improvements on the compensatory probe in the baseline VFSS maintained or increased those improvements across the same parameters after 4 weeks of rehabilitation. Additionally, 2 participants who did not show improvement on the compensatory probe, showed subsequent improvement at the post-rehabilitation VFSS, and 1 participant with compensatory improvement showed worse performance on a single parameter post-treatment. Overall, 4 of the 5 participants who completed the 4-week rehabilitative intervention showed improvement in 1 or more of the parameters investigated.

The parameters of interest in this study were selected based on hypothesized physiological and functional relations. Longer time-to-LVC is thought to contribute to penetration-aspiration, while reduced pharyngeal constriction is thought to contribute to post-swallow residue,^32^ both recognized as key components of dysphagia in PD. As shown in figures 1a-b and 2a-b, this study provides some support for the idea that time-to-LVC is a key mechanistic parameter underlying safe swallowing. The strong majority of observed improvements in Penetration-Aspiration Scale scores (both compensatory and rehabilitative) occurred in the context of corresponding improvements in time-to-LVC. Of course, there were also participants whose baseline PAS scores did not reflect any impairments, and some of these individuals also showed improvements in time-to-LVC. In terms of deterioration in swallowing, some participants were observed to evolve from a baseline PAS score of 1 to 2. It is important to note here that although PAS scores of 1 and 2 are known to occur in healthy adults, scores of 2 are less common than scores of 1.^29^ Based on this, scores of 2 have been considered atypical and heading in the direction of deterioration (but not reflective of serious clinical concern) in this study.

However, the story is not so clear with respect to changes in swallowing efficiency. Figures 1c-d and 2c-d do not show a close correspondence between improvements in total pharyngeal residue and improvements in PhAMPC. Indeed, in several cases, these 2 parameters showed opposite directions of change in the same participant. These patterns bring into question the presumed relation between pharyngeal constriction and residue and suggest that there may be other mechanisms at play. One factor that may be relevant here lies in the instructions that were used when teaching the ES. We chose to use a tongue-pressure emphasis technique, instructing participants to push-off hard against the anterior palate with their tongues when initiating an ES. This particular technique may have different effects and yield different results from ESs where the instructions emphasize greater pharyngeal squeeze or mental imagery of swallowing a large item such as a whole grape.

The heterogeneous findings in our study may be attributable to variations in individual participant responsiveness to intensive therapy. This may have been influenced by a variety of factors, including baseline oral intake status, time since PD onset, and PD severity. Although we attempted to recruit a homogeneous sample, with inclusion criteria mandating the presence of specific physiological impairments, the resulting sample was quite heterogeneous in nature. Despite these differences, the protocol intensity, duration, and frequency were held constant across all participants; individualizing the treatment protocol might have shown different outcomes. Future studies should explore the hypothesized mechanistic-functional relations between swallowing parameters, across a wider range of bolus textures, considering additional physiological mechanisms, which may also affect swallowing efficiency.

### Study limitations

This study was not without limitations. First, this study is a case series with a small sample size; therefore, caution is warranted when interpreting the results. A case series design was chosen given expected heterogeneity among participants, and given the goal of detecting improvements in swallowing safety/efficiency in a pilot study. However, in this case, where mixed results were scattered across all parameters in all 8 participants, it is challenging to derive conclusions regarding cause and effect relations and there is a risk of over-interpretation.

Second, we only studied participants who were exposed to the intervention. We acknowledge that comparison to a no-treatment control group would be needed to make clear inferences regarding intervention effects, The potential for spurious findings in a small case series justifies a larger, well-powered evaluation. Third, in order to summarize results across multiple repetitions for each task and consistency, the “worst” values per task and consistency condition were captured for each participant. This approach is common in dysphagia clinical practice, particularly for the Penetration-Aspiration Scale, which has categorical rather than interval properties.^33^ However, it is important to acknowledge that the convention of using worst scores may bias the analysis and does not account for the variation or frequency of specific scores seen during a VFSS. Fourth, previous literature suggests significant correlations between patient-reported subjective experience of dysphagia and PD disease severity,^13^^,^^34^, ^35^, ^36^, ^37^ and between objective measures of dysphagia and disease severity.^13^^,^^35^, ^36^, ^37^, ^38^ In this study, we asked patients to report swallowing-related quality of life using the Munich Dysphagia Test pre-intervention. This did not reveal correlations with the baseline VFSS measures of dysphagia or with subsequent improvement/deterioration. In the future, asking the patients to complete the questionnaire both pre- and post-treatment would allow for a valuable comparison of perceived change compared with change on objective VFSS measures. Finally, although a largely virtual intervention protocol can be beneficial when working with a neurodegenerative population, the possibility of variability in compliance to the protocol during home practice sessions exists. In our study, this was measured through patient/caregiver reports (homework logs), which were returned at the end of the intervention period. In the future, compliance and home session fidelity should be examined via home visits, videotaped sessions, or automatic logging on biofeedback devices.

## Conclusions

Our findings highlight the heterogeneous response to using the ES as both a compensatory and rehabilitative technique in people with PD. No consistent, systematic trends were identified in the direction of improvement or deterioration across penetration-aspiration scale scores, time-to-LVC, pharyngeal residue, or PhAMPC. This study points to the need for much larger sample sizes in order to confidently ascertain group-level benefits of the ES maneuver reinforced with the use of biofeedback, as a therapeutic resource in the rehabilitation of oropharyngeal dysphagia in people with PD.

## Appendix 1

### Additional details regarding study methods

#### Videofluoroscopy

Videofluoroscopic swallowing studies (VFSS) examinations were conducted at baseline and after the intervention protocol. These examinations were performed at 30 frames/s and included boluses of thin and mildly thick liquids as defined by the International Dysphagia Diet Standardization Initiative framework (www.iddsi.org). The barium stimuli were prepared using Bracco E-Z-Paque powdered barium in 20% w/v barium concentration and thickened with a gum-based thickening agent (Nestlé Resource ThickenUp Clear). Boluses were served in separate 4-oz cups, each containing 40 mL of liquid. To maximize ecological validity, participants were instructed to take a comfortable sip and to swallow when ready, ie, without a cue from the clinician. The baseline VFSS examination comprised 12 tasks: 3 repetitions of each consistency, swallowed with regular effort, and 3 repetitions of each consistency performed using the effortful swallow technique. The post-treatment VFSS comprised 6 tasks: 3 repetitions of each consistency, swallowed with regular effort.

#### Videofluoroscopy rating

The VFSS recordings for each bolus at each examination were relabeled with an alphanumeric code and assigned to 2 trained raters for independent rating using ImageJ software (https://imagej.nih.gov/ij/download.html) according to the Analysis of Swallowing Physiology: Events, Kinematics and Timing for Use in Clinical Practice Method (https://steeleswallowinglab.ca/srrl/). This standard operating procedure for rating begins by counting the number of swallows for each bolus, and scoring swallowing safety on each of those swallows using the Penetration-Aspiration Scale (PAS).^1^ The method continues by identifying the integrity of laryngeal vestibule closure (LVC) seen (complete/incomplete) and documenting the first-frames of hyoid burst onset, most-complete LVC, and of any penetration-aspiration events on the initial swallow of the bolus. The parameter time-to-LVC is then calculated as the difference, in frames, between hyoid burst and most-complete LVC, and converted to milliseconds based on a frame rate of 30 frames/second. Additionally, pixel-based measures of total pharyngeal residue and maximum pharyngeal constriction are made using the ImageJ area tracing tool and normalized to an anatomical scalar defined as the length of the cervical spine between the anterior inferior corners of the C2 and C4 vertebrae. All measures were made in duplicate and examined for inter-rater agreement. Exact agreement was required for number of swallows, PAS scores, and judgments of LVC integrity. Pre-specified thresholds for inter-rater difference tolerance were set at >3 frames for frame identification, and >2.4 %(C2-4)^2^ for area measures. In cases where inter-rater disagreement fell below these thresholds, rules were followed to select the earliest of the 2 candidate frames and the smaller of the 2 candidate area measures. In cases where inter-rater disagreement exceeded the pre-specified thresholds, a consensus meeting was convened for review and resolution, with a third rater present.

#### Intervention protocol

The 4-week intervention program involved two 30-minute sessions of ES practice daily, 5 days per week. Practice was supervised on a reducing schedule (week 1: 5 sessions; week 2: 4 sessions, etc), via secure videoconference using Microsoft Teams. Intervention involved biofeedback provided using the IOPI, which was loaned to each participant for home use. Each session included

• 15 regular saliva swallows with the IOPI bulb in place.
• 15 effortful saliva swallows with the IOPI bulb in place.
• 15 effortful swallows of mildly thick liquid without the IOPI bulb in place, for generalization.

A homework log was provided to each participant to document the number of tasks practiced in each session In addition, participants recorded tongue pressure values as displayed on the IOPI device after regular saliva swallows and effortful swallows, respectively. The homework logs were used to track compliance, alongside the supervised sessions.

A supply of thickener was provided to each participant for use in home practice sessions.

References

1. Rosenbek JC, Robbins JA, Roecker EB, Coyle JL, Wood JL. A penetration-aspiration scale. Dysphagia 1996;11:93-8.
